# All dielectric highly efficient achromatic meta-lens using inverse design optimization

**DOI:** 10.1038/s41598-023-45231-y

**Published:** 2023-11-01

**Authors:** Abdullah Maher, Mohamed A. Swillam

**Affiliations:** Department of Physics, The American University in Cairo, New Cairo, 11835 Egypt

**Keywords:** Optical materials and structures, Metamaterials

## Abstract

This work presents a high-efficiency achromatic meta-lens based on inverse design with topology optimization methodology. The meta-lens design with high numerical aperture values (NA = 0.7, NA = 0.8, and NA = 0.9) optimized along wavelength range starts from 550 to 800 nm, then the direct solver along the full extended wavelength band from 400 to 800 nm that applied to the final optimized structures under the three conditions of the high numerical apertures have high focusing efficiency for the all conditions. The optimization problem is based on Kreisselmeier–Steinhauser (k-s) objective function, leading to approximately stable response over the broadband bandwidths of the three designs.

## Introduction

Photonics devices are becoming a significant aspect of future technology since it relates to the synthesis, manipulation, and detection of light related to practical applications where the polarity of the light is vital^1–3^. It’s a major potential for designing and manufacturing devices, systems, and integrated circuits for applications in high-speed data transmission, enhancing sensing and imaging photonic technology promises orders of magnitude speed gains while consuming less power^4–7^. The optical performance of the photonics device is accessible by sweeping all the possible solutions where the higher order of the degree of freedom (DOF) requires a large simulation time, so the interest in satisfactory results and simulation time efficiency inverse design methodology employed, which depended mainly on the iterative optimization algorithms. In principle, the inverse design approaches require a clear definition of the objective function and the design of constraints. These constraints can be related to fabrication limitations, cost, and total footprint. The formulation of these parameters as an optimization problem is performed to achieve the optimal solution and the required optimal parameters^8–10^. The first one is shape optimization^11^ which starts with a suitable guess and perturbs the boundaries such that some figures of merit (FOM) are optimized. The second one is called the topology optimization^12–14^, which continuously varies the geometry yielding on optimal topology and shape that achieve the some FOM. In our work, we use inverse design with topology optimization to design a high-efficiency optical element, and our work relies on significant optical element meta-lens^15^. Meta-lens technology is gaining popularity due to its numerous uses in many applications^7,16^ such as polarization imaging systems, phase imaging systems, light field cameras, and solar energy harvesting. In the case of designing conventional or traditional meta-lens, there are two methods for dispersion elimination. The first method is called the panchayat man-berry phase (geometric phase)^17^ by changing the orientation of the meta-unit where the geometric phase operates under circularly polarized incidence. The second one is by controlling the resonance of the generally fluctuating^15^. The meta-lens based on the two designed is efficient in case of simple functionality in a single wavelength, but in many cases with complex functionality the forward or conventional design is not efficient such as the high efficiency under the high numerical aperture (NA) value^14^, in such this design and optimization challenge an inverse solution would be far more adept, so we use inverse design with topology optimization to produce broadband focusing efficiency with high NA meta-lens^18,19^. We demonstrated three designs based on three high numerical aperture conditions (NA = 0.7, NA = 0.8, and NA = 0.9) by observing the results with some parameters such as focusing efficiency, full-width half maximum (FWHM) and the field distribution along the design simulation area. Despite there is previous work studied the focusing efficiency of the meta-lens under high numerical aperture conditions^14^, The meta-lens design presented in this paper under the three numerical aperture conditions optimized along the wavelength band 250 nm starting from 550 to 800 nm, then solved directly along 400 nm starting from 400 to 800 nm and this wavelength range has potential application in microscopes, lithography machines and color display imaging. Due to the number of constraints (wavelengths) in the optimization problem, we need to reduce it by aggregation objective function. A maximum or a minimum value function is an obvious choice for these constraints aggregated, but both functions are not differentiable and inefficiently integrated with gradient-based design optimization, so the optimization problem depends on smooth estimators called the Kreisselmeier–Steinhauser (k-s)^20^. In gradient-based optimization, the k-s is an extensively used constraints aggregation methodology and has been applied in many applications, especially in civil construction design optimization^21^. In our design, the formulation of the optimization problem for the inverse design band from 550 to 800 nm depends on the Kreisselmeier–Steinhauser (k-s) objective function, where it targets 250 nm along the wavelengths band, but the design challenge in the topology optimization is related to the limitation of the spatial oscillation of the design field, so the design field is applied to the standard filtering and thresholding Heaviside function to recover between the two design materials. Titanium dioxide (${\mathrm{Tio}}_{2}$) is selected for all meta-lens design because it’s optically clear in the design band (400 nm: 1200 nm) and has excellent manufacturability in the nanoscale. Finally, the focusing efficiency of the meta-lens along the full band (400 nm: 800 nm) for the three numerical aperture conditions reaches a maximum value of 65.14%, 59.47%, and 53.21% for NA values 0.7, 0.8, and 0.9, respectively and the final optimized structure for the three designs are suitable for the fabrication process.

## Methods

### Working principle

**Figure 1:**
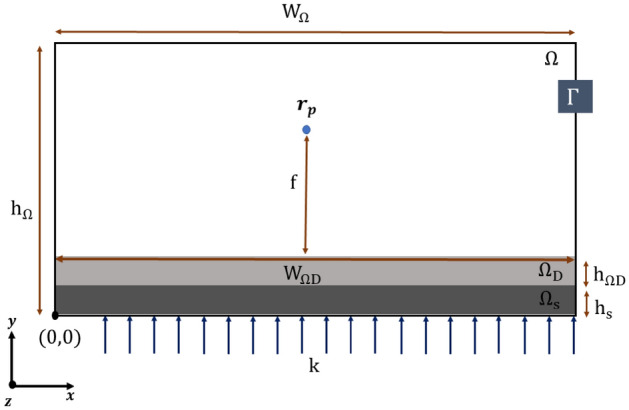
The meta-lens design and the boundary condition.

The Fig. 1 shows the boundaries Γ which subjected to the first order absorbing boundary conditions.

1 $$n\nabla {E}_{z}\left(r\right)=-ik{E}_{2}\left(r\right), r\in\Omega .$$

where n denotes the surface normal and i the imaginary unit. The model in Fig. 1 represents the meta-lens design consisting of the model domain Ω of height ${h}_{\Omega }$, which consists of substrate height ${h}_{\mathrm{s}}$, optimized region height ${h}_{\mathrm{\Omega D}}$, and remained height for air. The simulation width is ${w}_{\Omega }$, and the design width is ${w}_{\mathrm{\Omega D}}$. The focus point is ${r}_{p}$ which is determined then the field strength is determined to obtain the objective function $\Phi$, called the figure of merit (FOM). All dimensions of the design in Fig. 1 are listed in Table 1. The models are discretized by finite element method (FEM)^12^.

**Table 1:** Values for quantities in Fig. 1.

${h}_{\Omega }$	${w}_{\Omega }$	${w}_{\mathrm{\Omega D}}$	${h}_{\mathrm{\Omega D}}$	${h}_{\mathrm{s}}$
$8 \mu m$	$6 \mu m$	$6 \mu m$	$0.25 \mu m$	$0.25 \mu m$

The focal length $f$ and the focus point ${r}_{p}$ depending mainly on the numerical aperture condition^13^, and numerical aperture of the meta-lens can be calculated from:

2 $$\mathrm{NA}=\mathrm{sin}\left(\mathrm{arctan}\left(\frac{{w}_{{\Omega }_{D}}}{2f}\right)\right),$$

where the values of $f$ respected to the NA conditions are observed in Table 2.

**Table 2:** The values of $f$ respected to the NA condition.

$NA$	$0.7$	$0.8$	$0.9$
$f$	$3 \mu m$	$2.1 \mu m$	$1.4 \mu m$

Material interpolation algorithms are used in density-based topology optimization to relate a change in the design field to a change in the local spatial material property in the physical model problem^22^. The relation between refractive index $\eta$, extinction coefficient $k$, and electric permittivity ${\varepsilon }_{r}$ is given by:

3 $${\varepsilon }_{r}=\left({\eta }^{2}-{k}^{2}\right)-2i\eta k .$$

**Figure 2:**
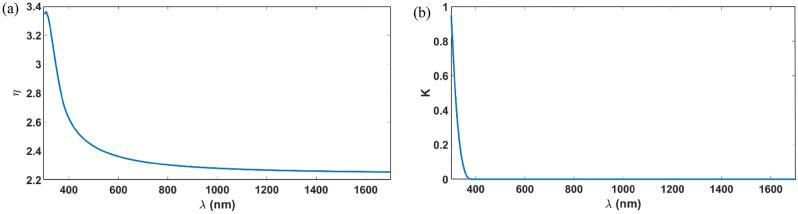
(**a**) The refractive index of Tio2; (**b**) The extinction coefficient of Tio_2_.

To formulate the non-linear interpolation scheme^22^:

4 $$\begin{array}{c}{\epsilon }_{r}(\eta (\rho ),\kappa (\rho ))=\left(\eta (\rho {)}^{2}-\kappa (\rho {)}^{2}\right)-i(2\eta (\rho )\kappa (\rho ))\\ \eta (\rho )={\eta }_{{\mathrm{M}}_{1}}+\rho \left({\eta }_{{\mathrm{M}}_{2}}-{\eta }_{{\mathrm{M}}_{1}}\right)\\ \kappa \left(\rho \right)={\kappa }_{{\mathrm{M}}_{1}}+\rho \left({\kappa }_{{\mathrm{M}}_{2}}-{\kappa }_{{\mathrm{M}}_{1}}\right) .\end{array}$$

where ${M}_{1}$ and ${M}_{2}$ denotes the two materials being interpolated, the interpolation parameters $\rho$ is varying from zero to one to relate between the two materials. The design is based on all ${\mathrm{Tio}}_{2}$ material as a high index material, and it’s interpolated with air as low index material. The measured data of the refractive index ($\eta$) and the extinction coefficient ($\kappa$) of the ${\mathrm{Tio}}_{2}$ that we depended on^23^ are shown in Fig. 2a and b, respectively.

The figure of merit $\phi$ as a function of the magnitude ${\left|E\right|}^{2}$ at the focal point ${r}_{p}$ is**:**

5 $$\phi \left(\rho (r),{r}_{p}\right)={\left|{E}_{z}\left(\rho (r),{r}_{p}\right)\right|}^{2}={E}_{z}{\left(\rho (r),{r}_{p}\right)}^{*}{E}_{z}\left(\rho (r),{r}_{p}\right) .$$

The optimization problem (Eq. 9) based on The Kreisselmeier–Steinhauser (k-s)^20^ objective function targets 250 nm of wavelength range simultaneously with the number of points (${N}_{\lambda }=20$). The k-s aggregation function is an alternative differentiable function to the max-mini function where the value of p overestimates the constraints minimum^20^. The formulation of FOM based on the k-s objective function can be written as:

6 $${\Phi }_{k-S}=\frac{-1}{p}\mathrm{ln}\left(\sum_{i=1}^{{N}_{\lambda }} {e}^{-p\left({\Phi }_{i}\left({E}_{z}\left({\lambda }_{i},{r}_{P},{\varepsilon }_{r}\left(\rho \left(r\right),{\lambda }_{i}\right)\right)\right)\right.}\right) .$$

The equality constraints of the optimization problem^12,13^ are related to the operator ${\mathcal{l}}_{EM },$ where the operator denotes applying the effect of the physical system to the state field for the excitation $F$. The solution to the optimization problem depends mainly on the interpolation parameters $\rho \left(r\right)$ to interpolate between the high index material (${\mathrm{Tio}}_{2}$) and the low index material (air). To limit the spatial oscillation of the design field a standard filtering is applied to the $\rho$ parameter over ${\Omega }_{\mathrm{D}}$ using the equation:

7 $$-{\left(\frac{{r}_{f}}{2\sqrt{3}}\right)}^{2}\nabla \widetilde{\rho }\left(\mathbf{r}\right)+\widetilde{\rho }\left(\mathbf{r}\right)=\rho \left(\mathbf{r}\right), {r}_{f}>0, r\in {\Omega }_{\mathrm{D}}.$$

where the ${r}_{f}$ is the filter radius. Then the filter is followed by thresholding using a smoothed approximation of the Heaviside function (Eq. 8) to recover a design between the Tio2 material and the background material (Air):

8 $$\stackrel{ \_\_}{\widetilde{\rho }}=\frac{\mathrm{tanh}(\beta \cdot \eta )+\mathrm{tanh}(\beta \cdot (\widetilde{\rho }-\eta ))}{\mathrm{tanh}(\beta \cdot \eta )+\mathrm{tanh}(\beta \cdot (1-\eta ))}, \beta \in [1,\infty [, \eta \in \left[\mathrm{0,1}\right].$$

where $\beta$ is the threshold strength and $\eta$ is the threshold level. The algorithm used to solve the design problem is MATLAB’s fmincon. The optimization parameters are listed in the Table 3. Where the ${n}_{iter}$ is the inner iteration taken to solve the optimization problem.

**Table 3:** The optimization parameters.

Parameter	$\eta$	$\beta$	${r}_{f}$(nm)	${n}_{iter}$	$\mathrm{p}$
Value	$0.5$	$5$	60	200	2

Finally, the optimization problem is formulated as:

9 $$\begin{array}{c}\underset{\rho }{max} \left(\frac{-1}{p}\mathrm{ln}\left(\sum_{\mathrm{i}=1}^{{N}_{2}} {e}^{-p\left({\Phi }_{\mathrm{i}}\left({E}_{\mathrm{z}}\left({\lambda }_{\mathrm{i}},{r}_{p},{\varepsilon }_{r}\left(\stackrel{ \_\_}{\widetilde{\rho }}(r),{\lambda }_{\mathrm{i}}\right)\right)\right.\right.}\right)\right)\\ \, {\text{s}}.{\text{t}}. \, \left.\ell_{EM}\left({E}_{z}\left({\lambda }_{\mathrm{i}},r\right),{\varepsilon }_{r}\left(\stackrel{ \_\_}{\widetilde{\rho }}(r),{\lambda }_{\mathrm{i}}\right)\right)=F\left(r,{\lambda }_{\mathrm{i}}\right)\right)\\ \left.{\varepsilon }_{r}\left(\stackrel{ \_\_}{\widetilde{\rho }}(r),{\lambda }_{\mathrm{i}}\right)=\left({\eta }^{2}\left(\stackrel{ \_\_}{\widetilde{\rho }}(r),{\lambda }_{\mathrm{i}}\right)-{k}^{2}\left(\stackrel{ \_\_}{\widetilde{\rho }}(r),{\lambda }_{\mathrm{i}}\right)\right)-2\mathrm{i}\eta \left(\stackrel{ \_\_}{\widetilde{\rho }}(r),{\lambda }_{\mathrm{i}}\right)k\left(\stackrel{ \_\_}{\widetilde{\rho }}(r),{\lambda }_{\mathrm{i}}\right)\right)\\ \eta \left(\stackrel{ \_\_}{\widetilde{\rho }}\left(r\right),{\lambda }_{\mathrm{i}}\right)={\eta }_{M1}\left({\lambda }_{\mathrm{i}}\right)+\rho \left(r\right)\left({\eta }_{M2}\left({\lambda }_{\mathrm{i}}\right)-{\eta }_{M1}\left({\lambda }_{\mathrm{i}}\right)\right)\\ k\left(\stackrel{ \_\_}{\widetilde{\rho }}\left(r\right),{\lambda }_{\mathrm{i}}\right)={k}_{M1}\left({\lambda }_{\mathrm{i}}\right)+\rho \left(r\right)\left({k}_{M2}\left({\lambda }_{\mathrm{i}}\right)-{k}_{M1}\left({\lambda }_{\mathrm{i}}\right)\right)\\ \begin{array}{c} \stackrel{ \_\_}{ \widetilde{\rho }}=\frac{\mathrm{tanh}(\beta \cdot \eta )+\mathrm{tanh}(\beta \cdot (\widetilde{\rho }-\eta ))}{\mathrm{tanh}(\beta \cdot \eta )+\mathrm{tanh}(\beta \cdot (1-\eta ))}, \\ -{\left(\frac{{r}_{f}}{2\sqrt{3}}\right)}^{2}\nabla \widetilde{\rho }\left(\mathbf{r}\right)+\widetilde{\rho }\left(\mathbf{r}\right)=\rho \left(\mathbf{r}\right) , \end{array}\\ 0\le \rho \left(r\right)\le 1,r\in {\Omega }_{\mathrm{D}}.\end{array}$$

The sensitivity of the k-s aggregation function (Eq. 10) respected to the design variable can be written as:

10 $$\frac{\partial {\Phi }_{k-s}(\rho )}{\partial \rho }=\frac{\sum_{i=1}^{{N}_{\lambda }} {e}^{-p\left({\Phi }_{i}(\rho )\right)}\frac{\partial {\Phi }_{i}(\rho )}{\partial \rho }}{\sum_{i=1}^{{N}_{\lambda }} {e}^{-p\left({\Phi }_{i}(\rho )\right)}}.$$

The gradient $\Phi$ respected to the design variables k $({\stackrel{ \_\_}{\widetilde{\rho }}}_{\mathrm{k}})$ is derived by the adjoint sensitivity method^12,24^:

11 $$\frac{\partial\Phi }{\partial {\stackrel{ \_\_}{\widetilde{\rho }}}_{\mathrm{k}}}=2\mathfrak{R}\left[{\lambda }^{T}\frac{\partial S}{{\stackrel{ \_\_}{\widetilde{\rho }}}_{\mathrm{k}}}{E}_{Z}\right] .$$

where $\lambda$ is a vector of nodal complex Lagrange multipliers and $\mathfrak{R}$ denotes the real part (Supplementary Information).

## Results

**Figure 3:**
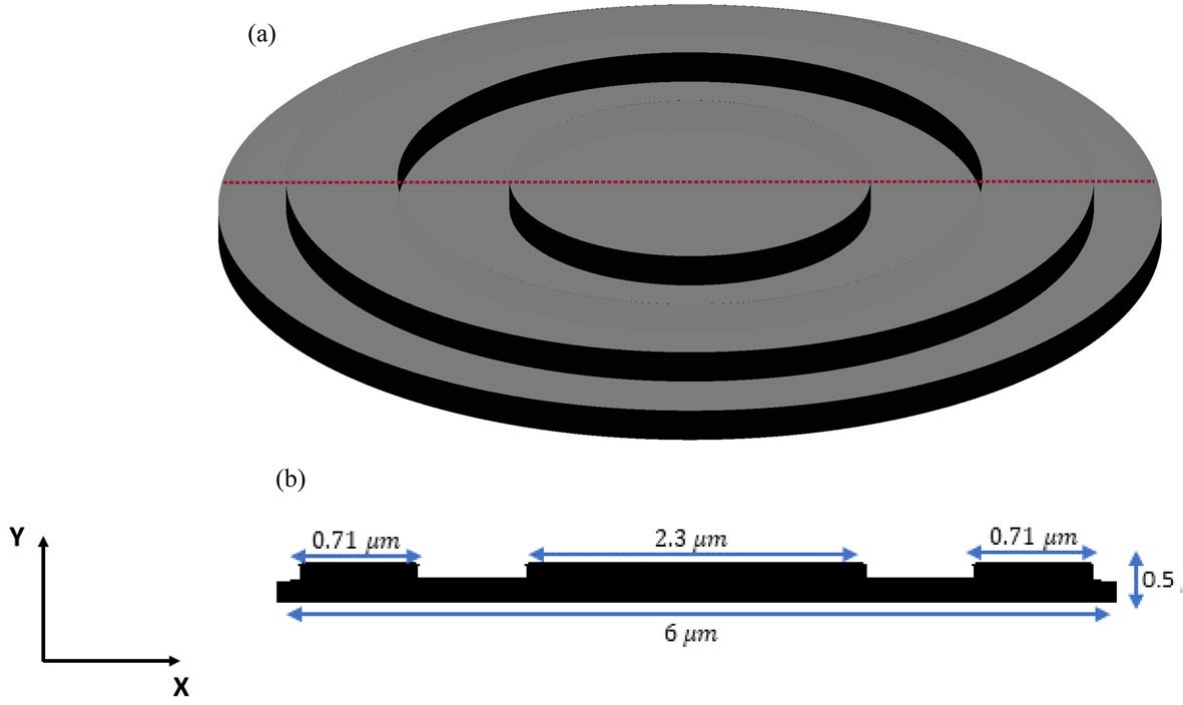
(**a**) 3D configuration of the meta-lens with NA = 0.7 (assuming radial symmetry). The cross section is taken along the red dashed line; (**b**) Cross section of the structure in the x–y plane, where the index represents the high index material (${\mathrm{Tio}}_{2}$) and the white represents the low index material (Air).

**Figure 4:**
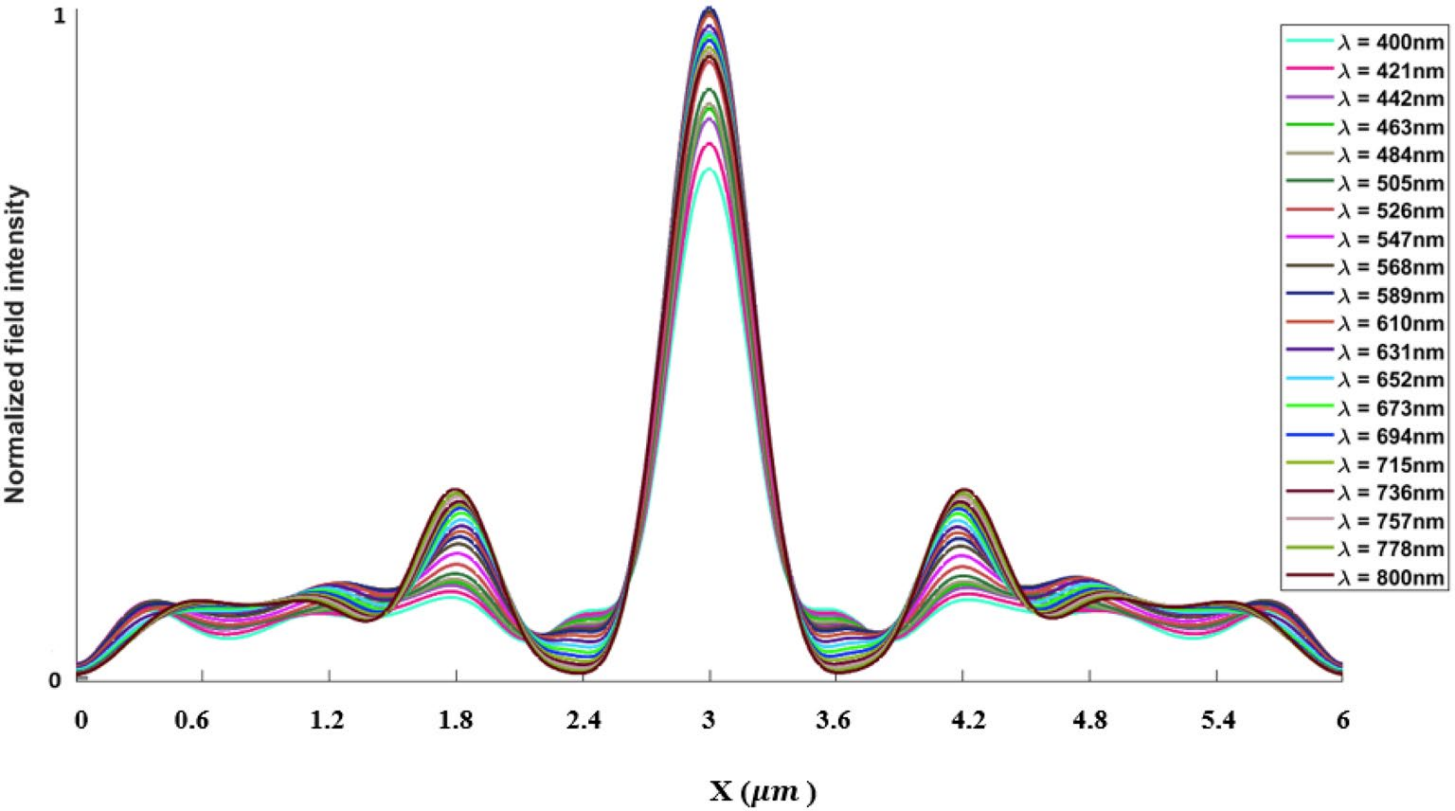
Normalized point spread function for meta-lens with NA = 0.7.

**Figure 5:**
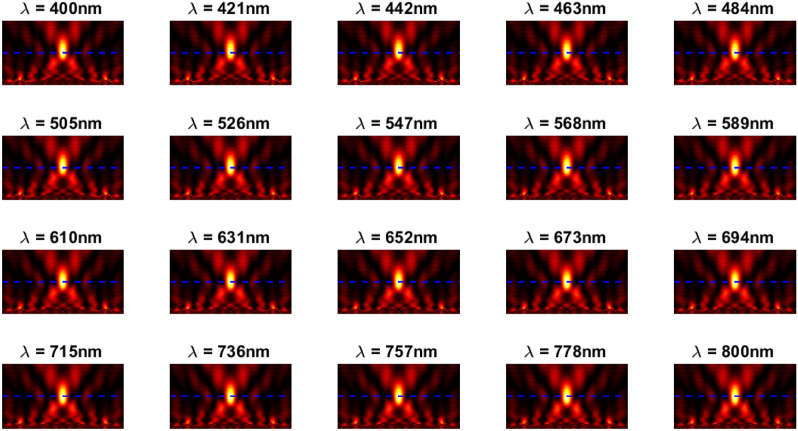
The electric field distribution along the X–Y plane for meta-lens with NA = 0.7.

**Figure 6:**
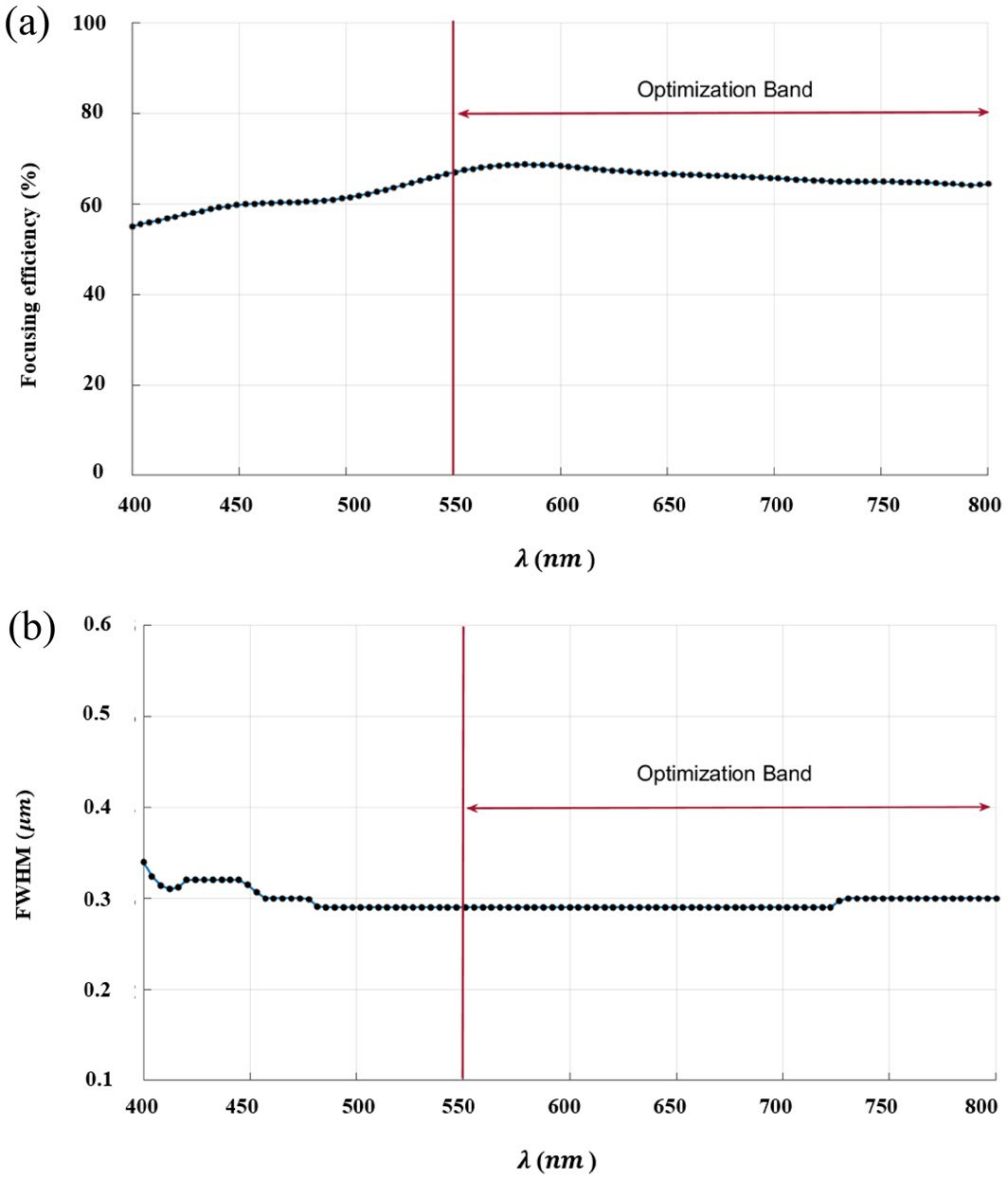
(**a**) The focusing efficiency of the meta-lens with NA = 0.7 along the wavelength range 400 nm to 800 nm. The efficiency ranges from 54.24 to 65.14% with an average 62.08%, while the focusing efficiency along the optimized band (550 nm to 800 nm) have a maximum of 68.45%, an average of 66.2% and minimum of 64.21%; (**b**) The full width half maximum of the meta-lens with NA = 0.7 along the wavelength range from 400 to 800 nm. The FWHM ranges from 290 to 340 nm with an average value of 296 nm, while the FWHM along the optimized band (550 nm to 800 nm) have a maximum of 300 nm, an average of 293 nm and minimum of 290 nm.

**Figure 7:**
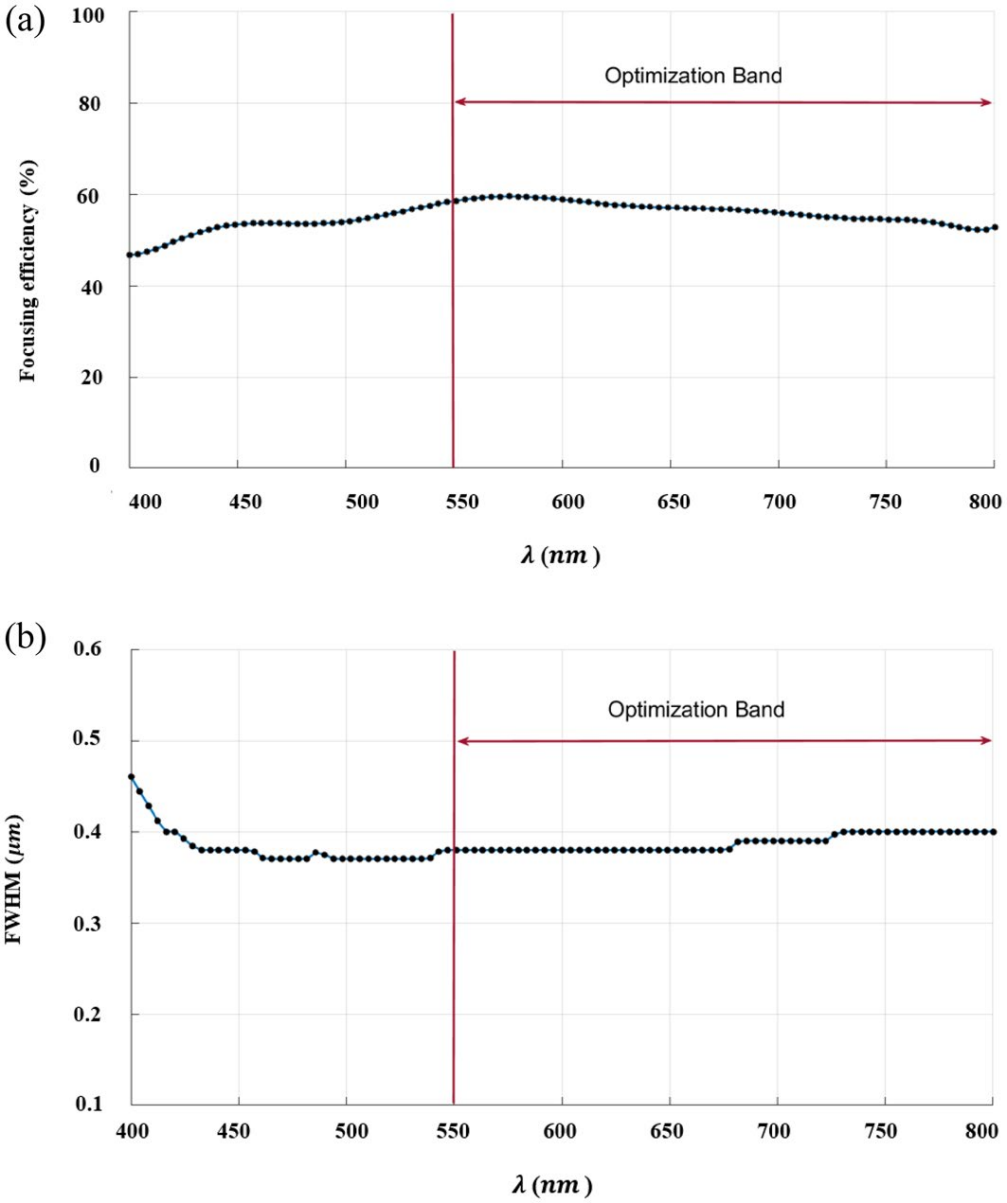
(**a**) The focusing efficiency of the meta-lens with NA = 0.8 along the wavelength range 400 nm to 800 nm. The efficiency ranges from 46.39% to 59.47% with an average 55.1%, while the focusing efficiency along the optimized band (550 nm to 800 nm) have a maximum of 59.27%, an average of 56.27%% and minimum of 52.49%; (**b**) The full width half maximum of the meta-lens with NA = 0.8 along the wavelength range from 400 to 800 nm. The FWHM ranges from 370 to 460 nm with an average value of 385 nm, while the FWHM along the optimized band (550 nm to 800 nm) have a maximum of 400 nm, an average of 388 nm and minimum of 380 nm.

**Figure 8:**
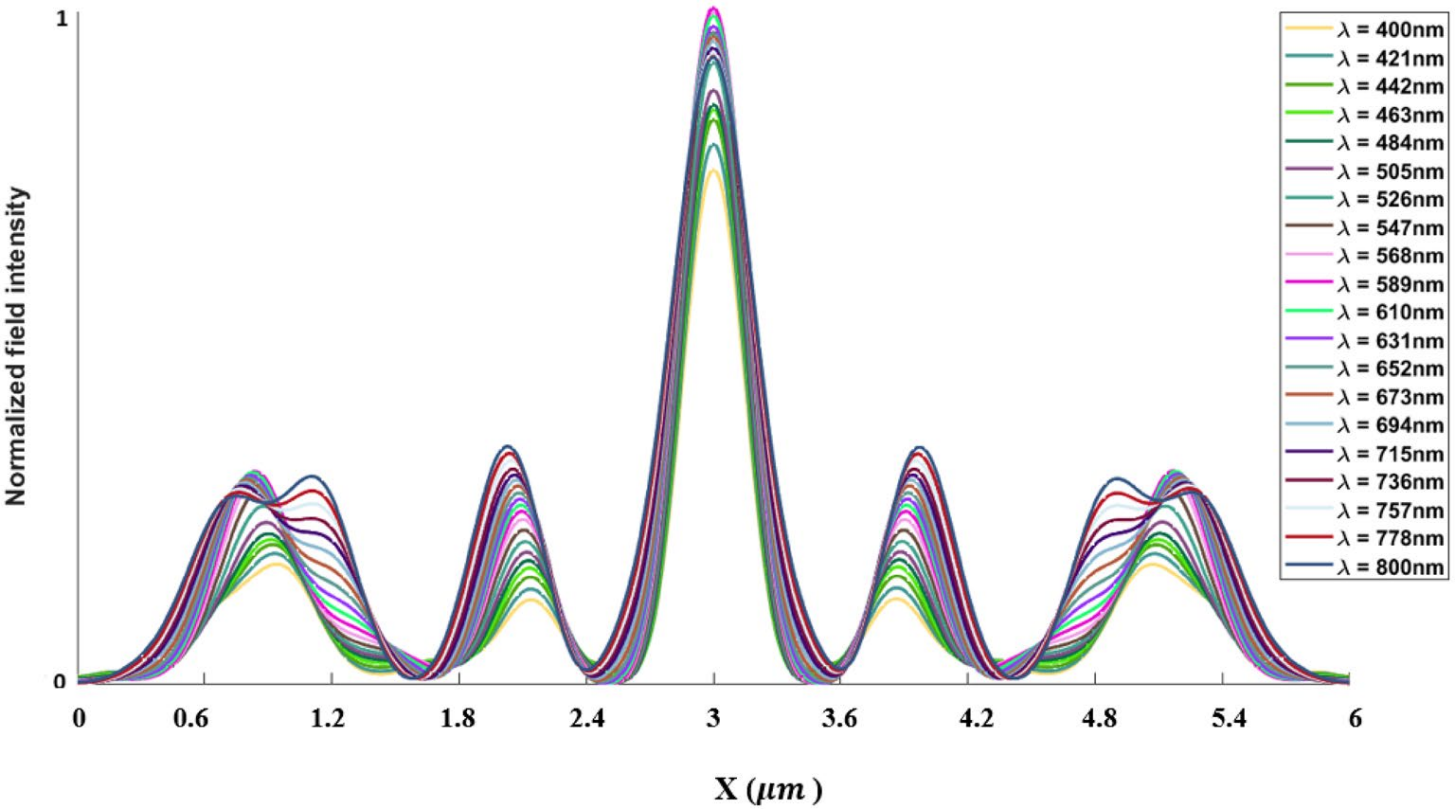
Normalized point spread function for meta-lens with NA = 0.8.

**Figure 9:**
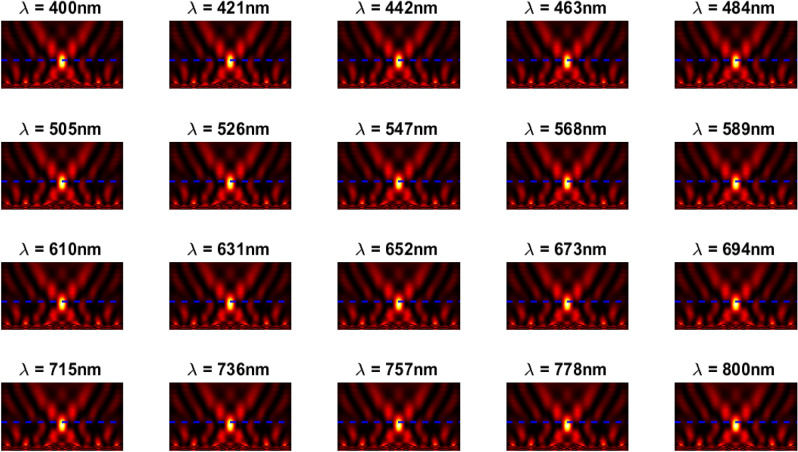
The electric field distribution along the X–Y plane for meta-lens with NA = 0.8.

**Figure 10:**
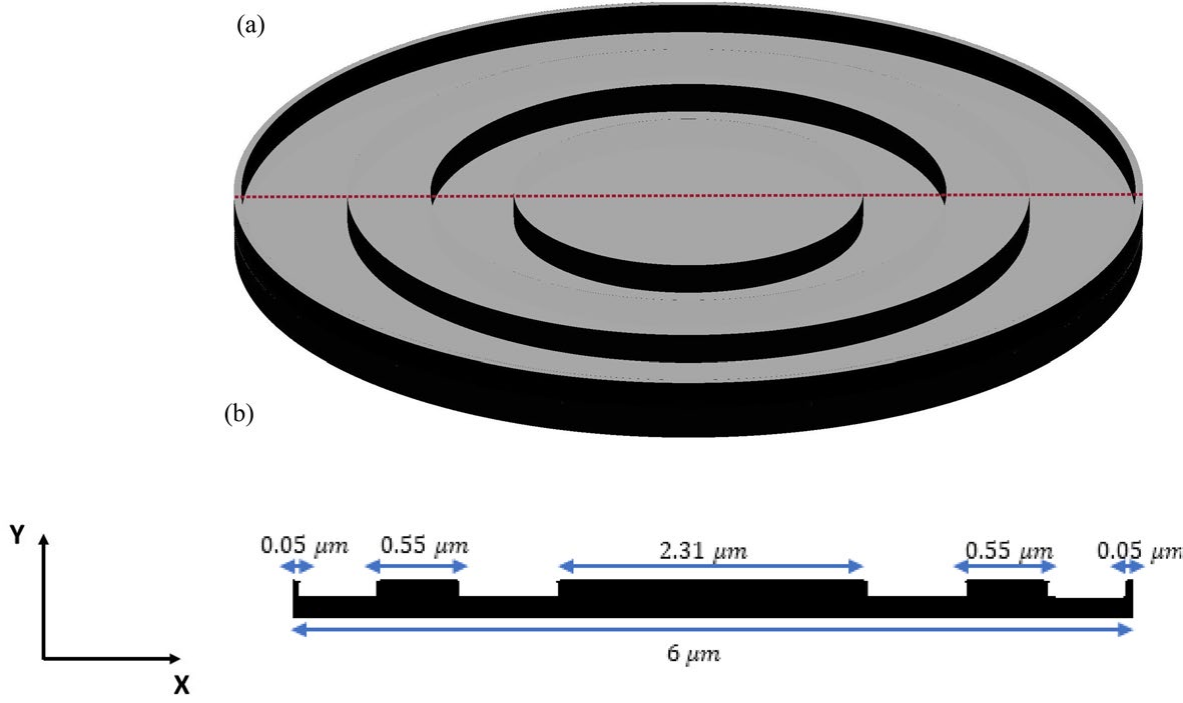
(**a**) 3D configuration of the meta-lens with NA = 0.8 assuming radial symmetry. The cross section is taken along the red dashed line; (**b**) Cross section of the structure in the x–y plane, where the black represents the high index material (${\mathrm{Tio}}_{2}$), and the white represents the low index material (Air).

The inverse design range starts from 550 to 800 nm. The final optimized structure under the three NA conditions (NA = 0.7, NA = 0.8 and NA = 0.9) resulting from the inverse design are solved directly from 400 to 800 nm with 100 wavelength points. To observe the results along the full wavelength range (400 nm to 800 nm), we need to define some illustrative parameters such as electric field distribution along the design area (x–y), the full width half maximum (FWHM), and finally, the focusing efficiency, where it’s calculated by the ratio of the power within the first minimum points to the incidence power^14^. For meta-lens under numerical aperture (NA = 0.7), the final binary structure is shown in Fig. 3, the points spread function for all wavelengths in Fig. 4 and from the electric field distribution along the (x–y) plane for all the wavelengths along the inverse range (Fig. 5) we can indicate from the focal plane (dashed blue line) that the focal lengths still within the depth of the focus point along the whole band (achromatic behavior). The focusing efficiency as observed in Fig. 6a has a maximum value of 65.14%. The average value is 62.08%, and the minimum value 54.24%, while the FWHM (Fig. 6b) has a maximum value of 340 nm and average value 296 nm. In the case of meta-lens with numerical aperture condition (NA = 0.8), the focusing efficiency on the inverse design band ranges from 46.39 to 59.47% with an average value of 55.1% (Fig. 7a), and The FWHM ranges from 370 to 460 nm with an average value of 385 nm (Fig. 7b). The point spread function (PSF), the final binarized structure and the electric field distribution are shown in Figs. 8, 9 and 10, respectively.

**Figure 11:**
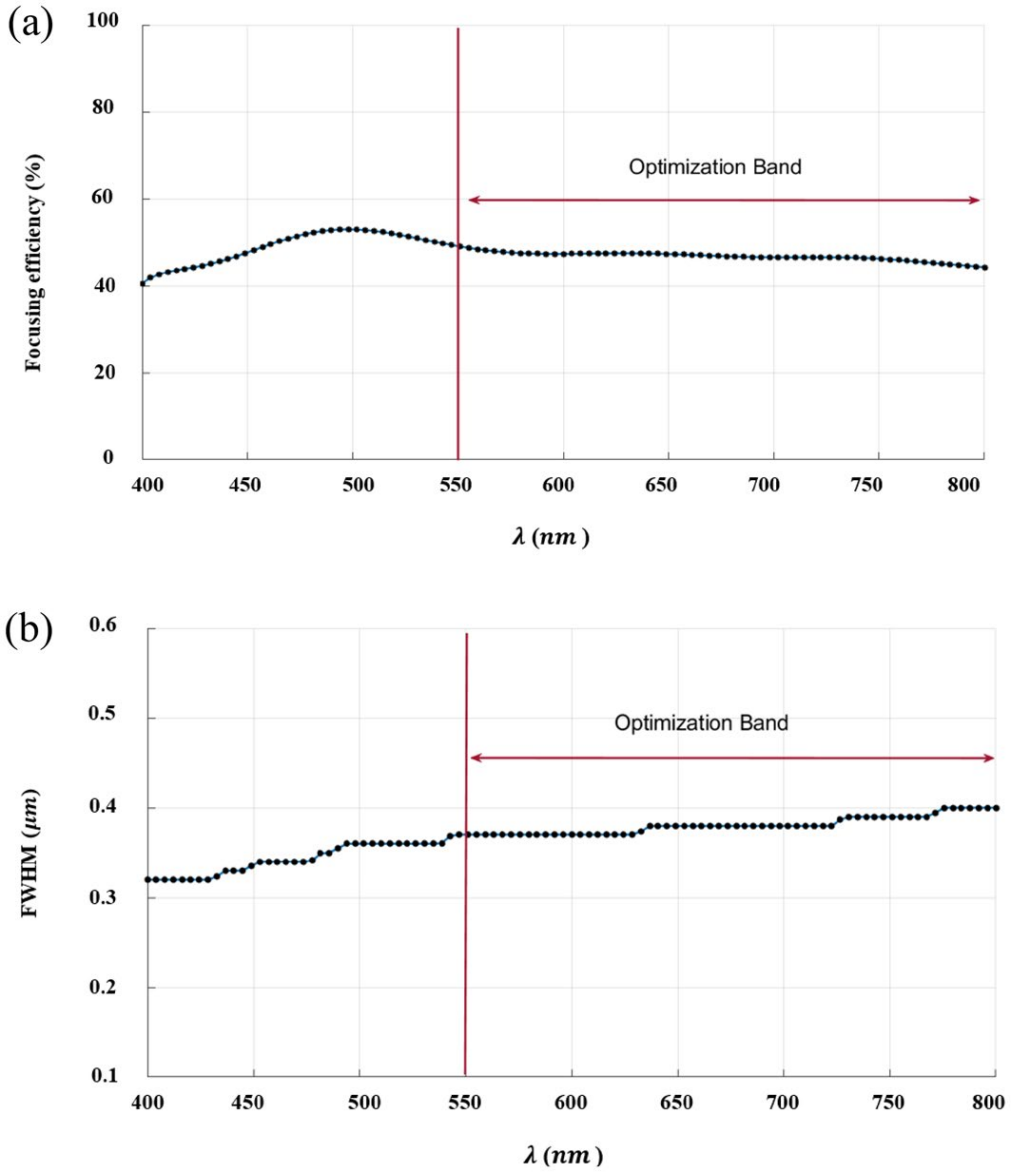
(**a**) The focusing efficiency of the meta-lens with NA = 0.9 along the wavelength range 400 nm to 800 nm. The efficiency ranges from 41.03% to 53.21% with an average 47.51%, while the focusing efficiency along the optimized band (550 nm to 800 nm) have a maximum of 47.9%, an average of 46.69% and minimum of 44.48%; (**b**) The full width half maximum of the meta-lens with NA = 0.9 along the wavelength range from 400 to 800 nm. The FWHM ranges from 320 to 400 nm with an average value of 366 nm, while the FWHM along the optimized band (550 nm to 800 nm) have a maximum of 400 nm, an average of 381 nm and minimum of 370 nm.

**Figure 12:**
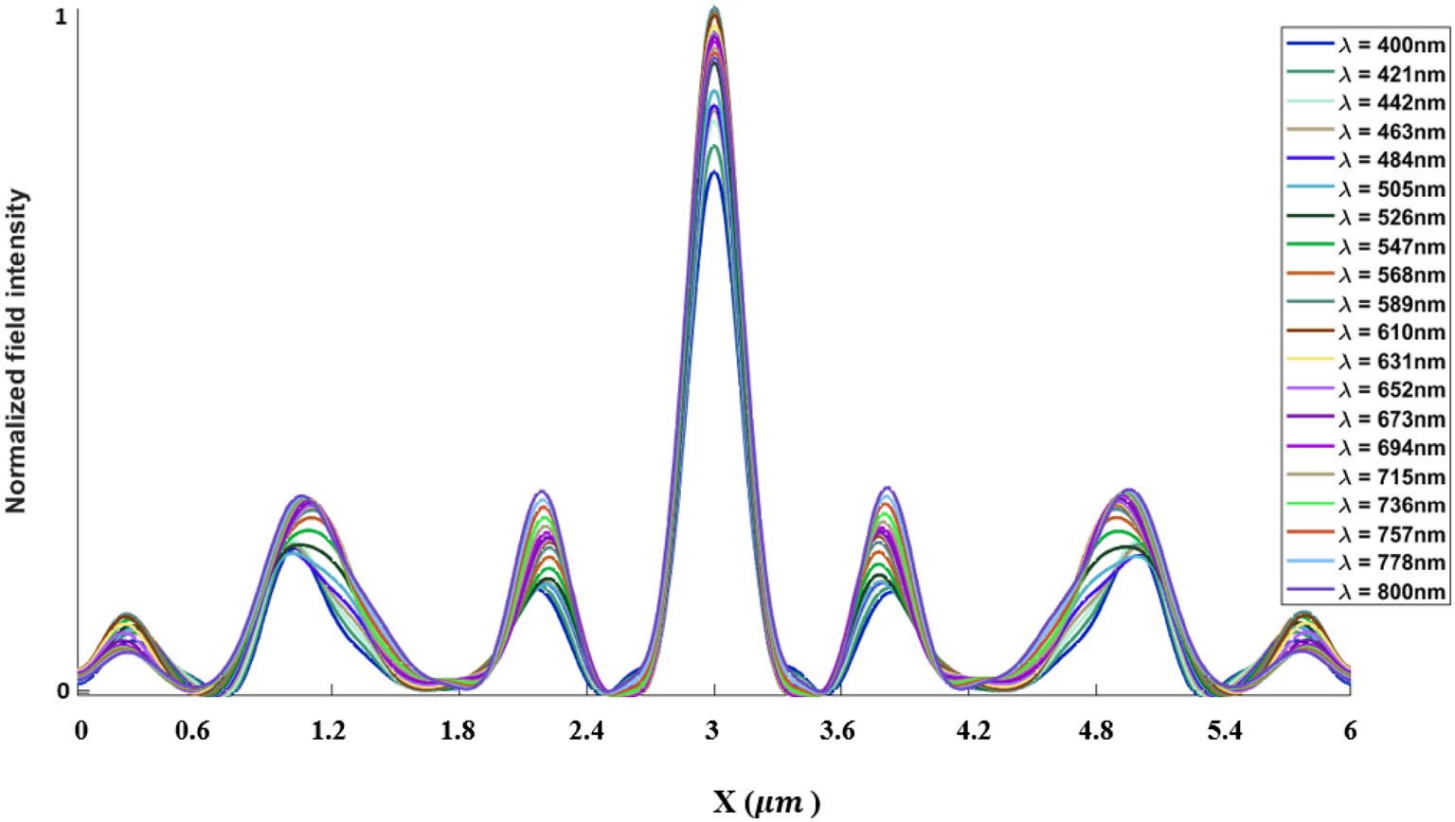
Normalized point spread function for meta-lens with NA = 0.9.

**Figure 13:**
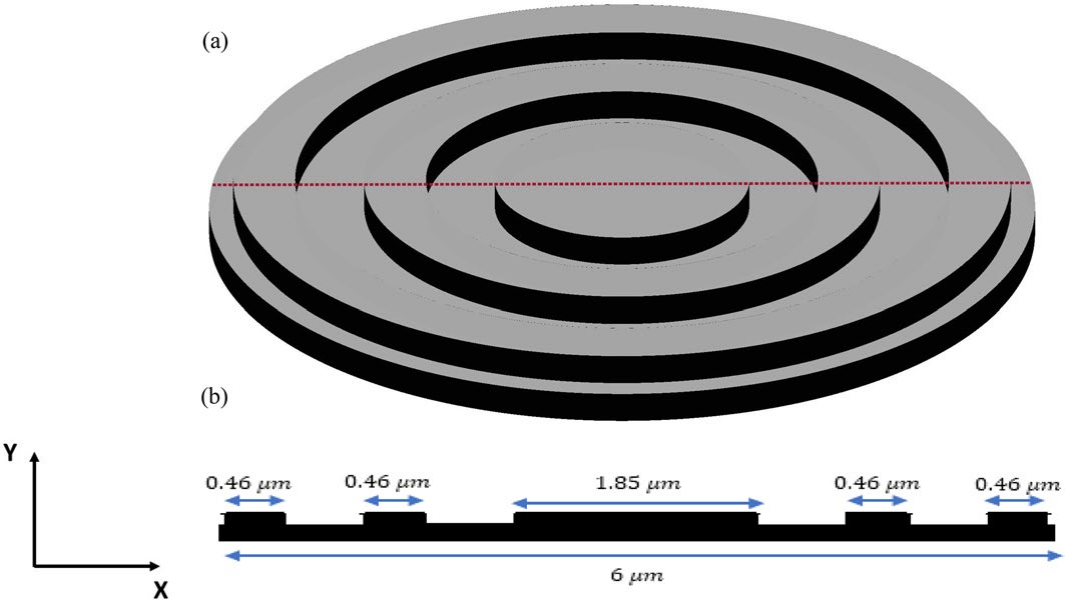
(**a**) 3D configuration of the meta-lens with NA = 0.9. The cross section is taken along the red dashed line; (**b**) cross section of the structure in x–y plane, where the black represents the high index material (${\mathrm{Tio}}_{2}$), and the white represents the low index material (Air).

**Figure 14:**
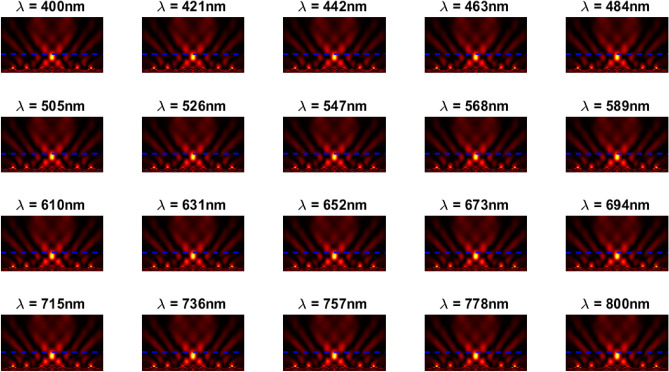
The electric field distribution along the X–Y plane for meta-lens with NA = 0.9.

The final design with a very high numerical aperture condition (NA = 0.9) reaches the maximum value of focusing efficiency 53.21% and an average value of 47.51% in Fig. 11a, while the FHWM reaches 400 nm as maximum and dropped to 320 nm as a minimum value (Fig. 11b), also the point spread function (PSF), the final binarized structure and the electric field distribution are shown in Figs. 12, 13 and 14, respectively.

Finally, for the all designs under the three NA conditions(NA = 0.7, NA = 0.8 and NA = 0.9) the average focusing efficiency are higher than the previously reported work in achromatic meta-lens^14,25,26^ especially the NA conditions for the three design are under very high NA, and the final structure design is friendly-lithography and suitable for fabrication process, also the results based on this inverse design model can have obvious impact on microscopy, biosensing, light field camera and imaging system where lead many applications now days.

## Conclusion

This paper presents a high efficiency broadband achromatic meta-lens based on inverse design with topology optimization. The design depends mainly on the ${\mathrm{Tio}}_{2}$ material in the wavelength range (400 nm: 800 nm). The results revealed that the focusing efficiency is very high for all numerical aperture conditions (NA = 0.7, NA = 0.8, NA = 0.9). The meta-lens leads to many applications and exhibits the potential for multiple optical system designs such as imaging system, biosensing applications, light field camera and spectroscopic system.

### Supplementary Information

Supplementary Information.

## Data Availability

The datasets used and/or analyzed during the current study available from the corresponding author on reasonable request.
