# Supplementary material for: All dielectric highly efficient achromatic meta-lens using inverse design optimization
Source: Sci Rep. 2023 Nov 1;13:18827. doi: 10.1038/s41598-023-45231-y (PMC10620238; doi:10.1038/s41598-023-45231-y)
Supplement: Supplementary file 1 — Supplementary Information. [file 41598_2023_45231_MOESM1_ESM.docx]

**Appendix. A: Adjoint sensitivity analysis**

The physical model is discretized using finite element method, so we can write the resulting equations of the linear system as:

$\mathbf{S}\left( \boldsymbol{\varepsilon}_{\boldsymbol{r}} \right)\mathbf{E}_{\boldsymbol{z}}\boldsymbol{=F}$. (1)

Where the $\mathbf{S}\left( \boldsymbol{\varepsilon}_{\boldsymbol{r}} \right)$ is the design dependent system matrix and $\mathbf{E}_{\boldsymbol{z}}$ is a vector of degrees of freedom for the electric field.

The gradient of the FOM with the design variable $\boldsymbol{\rho}_{\boldsymbol{k}}$ calculated as:

$\frac{\boldsymbol{d\Phi}}{\mathbf{d}{\overset{\boldsymbol{\_\_}}{\tilde{\boldsymbol{\rho}}}}_{\mathbf{k}}}\boldsymbol{=}\frac{\boldsymbol{\partial\Phi}}{\boldsymbol{\partial}\boldsymbol{E}_{\mathbf{z,}\mathfrak{R}}}\frac{\boldsymbol{\partial}\boldsymbol{E}_{\mathbf{z,}\mathfrak{R}}}{\boldsymbol{\partial}{\overset{\boldsymbol{\_\_}}{\tilde{\boldsymbol{\rho}}}}_{\mathbf{k}}}\boldsymbol{+}\frac{\boldsymbol{\partial\Phi}}{\boldsymbol{\partial}\boldsymbol{E}_{\mathbf{z,}\mathfrak{I}}}\frac{\boldsymbol{\partial}\boldsymbol{E}_{\mathbf{z,}\mathfrak{I}}}{\boldsymbol{\partial}{\overset{\boldsymbol{\_\_}}{\tilde{\boldsymbol{\rho}}}}_{\mathbf{k}}}$ . (2)

Where the $\boldsymbol{E}_{\mathbf{z,}\mathfrak{R}}$ and $\boldsymbol{E}_{\mathbf{z,}\mathfrak{I}}$ denotes the real and imaginary part of the electric field respectively. By adding zero the FOM twice,

$\tilde{\boldsymbol{\Phi}}\boldsymbol{=}\boldsymbol{\Phi}\boldsymbol{+}\boldsymbol{\lambda}^{\boldsymbol{T}}\left( \mathbf{SE}_{\boldsymbol{z}}\boldsymbol{-}\mathbf{F} \right)\boldsymbol{+}\boldsymbol{\lambda}^{\boldsymbol{\dagger}}\left( \mathbf{S}^{\boldsymbol{*}}\mathbf{E}_{\boldsymbol{z}}^{\boldsymbol{*}}\boldsymbol{-}\mathbf{F}^{\boldsymbol{*}} \right)$ . (3)

Where the $\boldsymbol{\lambda}^{\boldsymbol{T}}$ is the transpose of the Lagrange multipliers (adjoint variables). Then taking the derivative of $\tilde{\boldsymbol{\Phi}}$ with respect to the design variables ${\overset{\boldsymbol{\_\_}}{\tilde{\boldsymbol{\rho}}}}_{\mathbf{k}}$ the result is computed as:

$\begin{aligned} \frac{\boldsymbol{d}\tilde{\boldsymbol{\Phi}}}{\boldsymbol{d}{\overset{\boldsymbol{\_\_}}{\tilde{\boldsymbol{\rho}}}}_{\mathbf{k}}}\boldsymbol{=&}\frac{\boldsymbol{\partial\Phi}}{\boldsymbol{\partial}\mathbf{E}_{\boldsymbol{z,}\mathfrak{R}}}\frac{\boldsymbol{\partial}\mathbf{E}_{\boldsymbol{z,}\mathfrak{R}}}{\boldsymbol{\partial}{\overset{\boldsymbol{\_\_}}{\tilde{\boldsymbol{\rho}}}}_{\mathbf{k}}}\boldsymbol{+}\frac{\boldsymbol{\partial\Phi}}{\boldsymbol{\partial}\mathbf{E}_{\boldsymbol{z,}\mathfrak{I}}}\frac{\boldsymbol{\partial}\mathbf{E}_{\boldsymbol{z,}\mathfrak{I}}}{\boldsymbol{\partial}{\overset{\boldsymbol{\_\_}}{\tilde{\boldsymbol{\rho}}}}_{\mathbf{k}}} \\ \boldsymbol{&+}\boldsymbol{\lambda}^{\boldsymbol{T}}\left( \frac{\boldsymbol{\partial S}}{\boldsymbol{d}{\overset{\boldsymbol{\_\_}}{\tilde{\boldsymbol{\rho}}}}_{\mathbf{k}}}\mathbf{E}_{\boldsymbol{z}}\boldsymbol{+}\mathbf{S}\left( \frac{\boldsymbol{\partial}\mathbf{E}_{\boldsymbol{z,}\mathfrak{R}}}{\boldsymbol{\partial}{\overset{\boldsymbol{\_\_}}{\tilde{\boldsymbol{\rho}}}}_{\mathbf{k}}}\boldsymbol{+}\mathbf{i}\frac{\boldsymbol{\partial}\mathbf{E}_{\boldsymbol{z,}\mathfrak{F}}}{\boldsymbol{\partial}{\overset{\boldsymbol{\_\_}}{\tilde{\boldsymbol{\rho}}}}_{\mathbf{k}}} \right) \right) \\ \boldsymbol{&+}\boldsymbol{\lambda}^{\boldsymbol{\dagger}}\left( \frac{\boldsymbol{\partial}\mathbf{S}^{\boldsymbol{*}}}{\boldsymbol{\partial}{\overset{\boldsymbol{\_\_}}{\tilde{\boldsymbol{\rho}}}}_{\mathbf{k}}}\mathbf{E}_{\boldsymbol{z}}^{\boldsymbol{*}}\boldsymbol{+}\mathbf{S}^{\boldsymbol{*}}\left( \frac{\boldsymbol{\partial}\mathbf{E}_{\boldsymbol{z,}\mathfrak{R}}}{\boldsymbol{\partial}{\overset{\boldsymbol{\_\_}}{\tilde{\boldsymbol{\rho}}}}_{\mathbf{k}}}\boldsymbol{-}\mathbf{i}\frac{\boldsymbol{\partial}\mathbf{E}_{\boldsymbol{z,}\mathfrak{I}}}{\boldsymbol{\partial}{\overset{\boldsymbol{\_\_}}{\tilde{\boldsymbol{\rho}}}}_{\mathbf{k}}} \right) \right)\boldsymbol{.} \end{aligned}$ (4)

Collecting the two terms $\frac{\boldsymbol{\partial}\mathbf{E}_{\boldsymbol{z,}\mathfrak{R}}}{\boldsymbol{\partial}{\overset{\boldsymbol{\_\_}}{\tilde{\boldsymbol{\rho}}}}_{\mathbf{k}}}$ and $\frac{\boldsymbol{\partial}\mathbf{E}_{\boldsymbol{z,}\mathfrak{I}}}{\boldsymbol{\partial}{\overset{\boldsymbol{\_\_}}{\tilde{\boldsymbol{\rho}}}}_{\mathbf{k}}}$ and reducing the remaining terms yields

$\begin{aligned} \frac{\boldsymbol{d}\tilde{\boldsymbol{\Phi}}}{\boldsymbol{d}{\overset{\boldsymbol{\_\_}}{\tilde{\boldsymbol{\rho}}}}_{\mathbf{k}}}\boldsymbol{=&}\frac{\boldsymbol{\partial}\mathbf{E}_{\boldsymbol{z,}\mathfrak{R}}}{\boldsymbol{\partial}{\overset{\boldsymbol{\_\_}}{\tilde{\boldsymbol{\rho}}}}_{\mathbf{k}}}\left( \frac{\boldsymbol{\partial\Phi}}{\boldsymbol{\partial}\mathbf{E}_{\boldsymbol{z,}\mathfrak{R}}}\boldsymbol{+}\boldsymbol{\lambda}^{\boldsymbol{T}}\mathbf{S}\boldsymbol{+}\boldsymbol{\lambda}^{\boldsymbol{\dagger}}\mathbf{S}^{\boldsymbol{*}} \right) \\ \boldsymbol{&+}\frac{\boldsymbol{\partial}\mathbf{E}_{\boldsymbol{z,}\mathfrak{s}}}{{\overset{\boldsymbol{\_\_}}{\tilde{\boldsymbol{\rho}}}}_{\mathbf{k}}}\left( \frac{\boldsymbol{\partial\Phi}}{\boldsymbol{\partial}\mathbf{E}_{\boldsymbol{z,}\mathcal{S}}}\boldsymbol{+}\mathbf{i}\boldsymbol{\lambda}^{\boldsymbol{T}}\mathbf{S}\boldsymbol{+}\mathbf{i}^{\boldsymbol{\dagger}}\mathbf{S}^{\boldsymbol{*}} \right)\boldsymbol{+2}\mathfrak{R}\left( \boldsymbol{\lambda}^{\boldsymbol{T}}\frac{\boldsymbol{\partial S}}{{\overset{\boldsymbol{\_\_}}{\tilde{\boldsymbol{\rho}}}}_{\mathbf{k}}}\mathbf{E}_{\boldsymbol{z}} \right)\boldsymbol{.} \end{aligned}$ (5)

The elimination of the first two terms in eq.16 with the two derivates $\frac{\boldsymbol{\partial}\mathbf{E}_{\boldsymbol{z,}\mathfrak{R}}}{\boldsymbol{\partial}{\overset{\boldsymbol{\_\_}}{\tilde{\boldsymbol{\rho}}}}_{\mathbf{k}}}$ and $\frac{\boldsymbol{\partial}\mathbf{E}_{\boldsymbol{z,}\mathfrak{I}}}{\boldsymbol{\partial}{\overset{\boldsymbol{\_\_}}{\tilde{\boldsymbol{\rho}}}}_{\mathbf{k}}}$, the two parentheses must equal zero,

$\frac{\boldsymbol{\partial\Phi}}{\boldsymbol{\partial}\mathbf{E}_{\boldsymbol{z,}\mathfrak{R}}}\boldsymbol{+}\boldsymbol{\lambda}^{\boldsymbol{T}}\mathbf{S}\boldsymbol{+}\boldsymbol{\lambda}^{\boldsymbol{\dagger}}\mathbf{S}^{\boldsymbol{*}}\boldsymbol{=0,}\frac{\boldsymbol{\partial\Phi}}{\boldsymbol{\partial}\mathbf{E}_{\boldsymbol{z,}\mathfrak{S}}}\boldsymbol{+}\mathbf{i}\boldsymbol{\lambda}^{\boldsymbol{T}}\mathbf{S}\boldsymbol{+}\mathbf{i}\boldsymbol{\lambda}^{\boldsymbol{\dagger}}\mathbf{S}^{\boldsymbol{*}}\boldsymbol{=0 .}$ (6)

Multiplying the second equation by I, subtracting it from the first, and transposing it yields

$\frac{\boldsymbol{\partial\Phi}}{\boldsymbol{\partial}\mathbf{E}_{\boldsymbol{z,}\mathfrak{R}}}\boldsymbol{-}\mathbf{i}\frac{\boldsymbol{\partial\Phi}}{\boldsymbol{\partial}\mathbf{E}_{\boldsymbol{z,}\mathfrak{I}}}\boldsymbol{+2}\boldsymbol{\lambda}^{\boldsymbol{T}}\mathbf{S}\boldsymbol{=0\Leftrightarrow}\mathbf{S}^{\boldsymbol{T}}\boldsymbol{\lambda=-}\frac{\boldsymbol{1}}{\boldsymbol{2}}\left( \frac{\boldsymbol{\partial\Phi}}{\boldsymbol{\partial}\mathbf{E}_{\boldsymbol{z,}\mathfrak{R}}}\boldsymbol{-}\mathbf{i}\frac{\boldsymbol{\partial\Phi}}{\boldsymbol{\partial}\mathbf{E}_{\boldsymbol{z,}\mathfrak{I}}} \right)^{\boldsymbol{T}}\boldsymbol{.}$ (7)

When the requiring in eq. 18 is satisfied, the final expression of the eq.15 will be

$\frac{\partial\Phi}{\partial{\overset{\_\_}{\tilde{\rho}}}_{k}}=2\mathfrak{R}\left[ \lambda^{T}\frac{\partial S}{{\overset{\_\_}{\tilde{\rho}}}_{k}}E_{Z} \right].$ (8)

**Appendix. B: The convergence history**

Supplementary Fig.1 observes the convergence history of the normalized FOM respected to each wavelength in RBG color over the inverse design iteration. The FOM has a stable convergence until reaches the local optima and requires only 37 iterations.


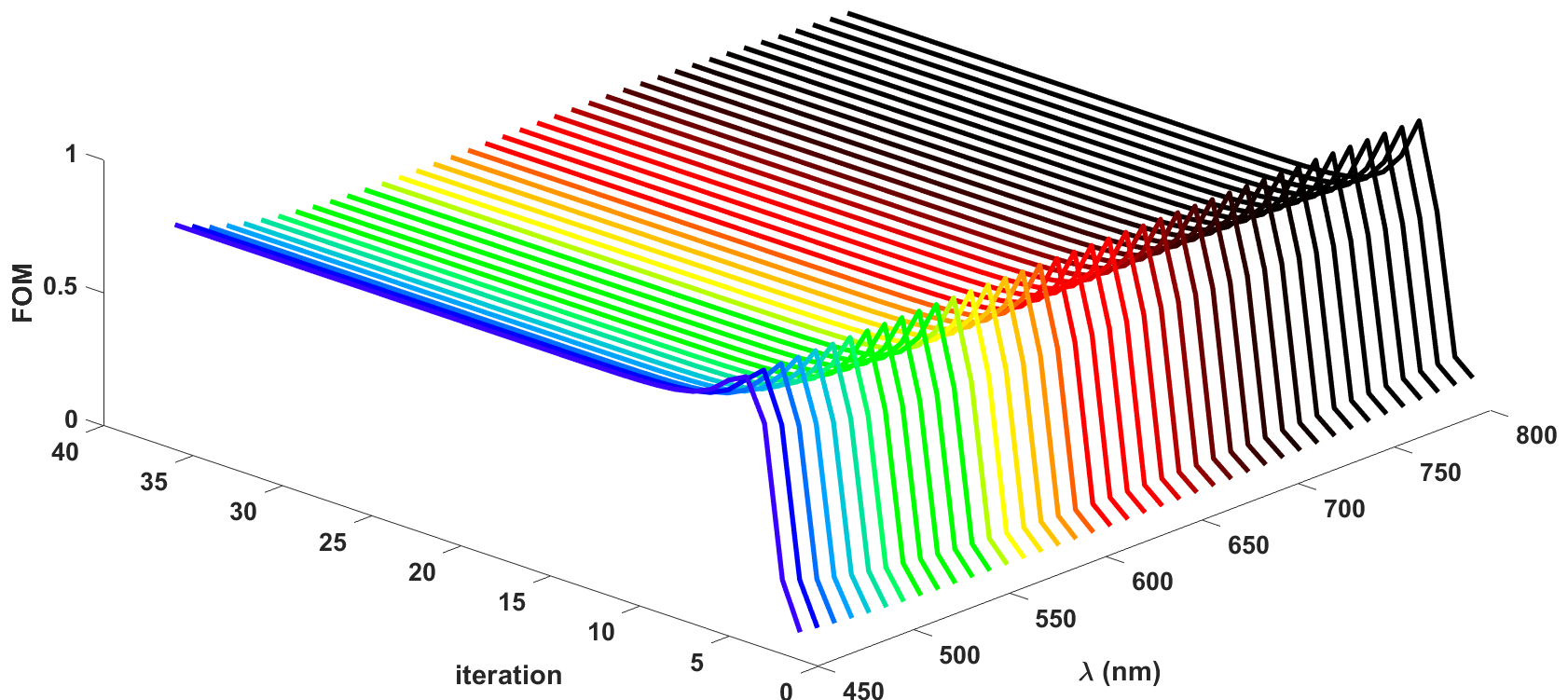


**Supplementary Figure1.**  The convergence history over the design iteration for meta-lens with NA = 0.7.
